# When Small Meets Smaller: Immune Modulation and Virulence Strategies in Insect–Bacteria Interactions

**DOI:** 10.3390/insects17050515

**Published:** 2026-05-19

**Authors:** Tommaso Bianchi, Maristella Mastore, Davide Banfi, Ameni Loulou, Silvia Quadroni, Maurizio F. Brivio

**Affiliations:** 1 Laboratory of Applied Entomology and Parasitology, Department of Theoretical and Applied Sciences (DiSTA), University of Insubria, 21100 Varese, Italy; tbianchi1@studenti.uninsubria.it (T.B.); maristella.mastore@uninsubria.it (M.M.); davidebanfiphd@outlook.it (D.B.); 2 Laboratory of Bio-Aggressors and Integrated Protection in Agriculture, Department of Plant Health and Environment, National Agronomic Institute of Tunisia, University of Carthage, Tunis 1082, Tunisia; loulouameni87@gmail.com; 3 Laboratory of Ecology, Department of Theoretical and Applied Sciences, University of Insubria, 21100 Varese, Italy; silvia.quadroni@uninsubria.it

**Keywords:** host–microorganism, entomopathogen, infection routes, immune pathways, immune priming, evasion strategies, alternative models

## Abstract

Insects represent powerful experimental systems for investigating host–microorganism interactions, providing valuable insights into bacterial pathogenicity, immune regulation, symbiosis, and antimicrobial discovery. This review examines the complex relationships between insects and bacteria, focusing on the mechanisms that control infection, immune activation, and microbial adaptation. Particular attention is given to the routes of pathogen entry and to the conserved innate immune pathways that coordinate host defenses, including the Toll, Imd, Duox, and Jak/Stat signaling cascades. The review illustrates how bacterial pathogens exploit toxins, immune evasion strategies, and metabolic adaptation to overcome host defenses, while insects rely on tightly regulated cellular and humoral responses, antimicrobial peptides, melanization, and microbiota-mediated homeostasis. Interactions between pathogenic and commensal bacteria in the insect gut are discussed in the context of immune tolerance, dysbiosis, and ecological adaptation. The dual role of bacterial virulence factors in both pathogenesis and symbiosis is highlighted through examples involving entomopathogenic bacteria such as *Photorhabdus* spp., *Xenorhabdus* spp., and *Bacillus thuringiensis*. In addition, the review summarizes the use of insect models, including *Drosophila melanogaster*, *Galleria mellonella*, *Bombyx mori*, and *Apis mellifera*, in experimental infections aimed at studying virulence mechanisms, host immune responses, and antimicrobial efficacy. Finally, multi-omic approaches, including transcriptomics, metabolomics, epigenomics, and single-cell technologies are discussed as transformative tools for dissecting host–microbe interactions at molecular and systems levels. Overall, insect–bacteria interactions emerge as dynamic and evolutionarily shaped systems in which immunity, metabolism, microbiota composition, and environmental factors are closely interconnected, offering important perspectives for both basic research and the development of sustainable biocontrol and antimicrobial strategies.

## Simple Summary

Insects are widely used as models to study interactions between bacteria and their hosts, including how bacteria cause disease and host immune responses. This review focuses on insect–bacteria relationships. It describes how factors such as infection routes, bacterial virulence, and insect immunity influence infection outcomes, highlighting key immune pathways. The review also discusses interactions between pathogenic and non-pathogenic bacteria, including effects on immune balance and dysbiosis. Finally, it presents key insect-pathogenic bacteria, describing how different bacterial strains are used with insect models to study immunity, biological control, and the efficacy of antimicrobial compounds, including recent advances in integrated multi-omic analyses.

## 1. Introduction

This review aims to provide an overview of the processes that regulate interactions between bacteria and their insect hosts. In particular, it aims to present an integrated perspective on the main mechanisms underlying host–microorganism interactions, examining their environmental implications, also in relation to biocontrol strategies. Furthermore, the physiological aspects that characterize the progression of infectious processes in the biomedical field are considered, as well as the potential applications of invertebrate models in the research and development of new antimicrobial drugs.

Insects, with an estimated 5.5 million extant species [1], dominate terrestrial biodiversity and interact continuously with a diverse microbial spectrum. In addition to their ecological importance, insects are increasingly studied for eco-sustainable pest control strategies [2,3] and for physiological traits that make them valuable models in immunological and pharmacological research [4,5,6]. Some species considered harmful to agroecosystems and urban green areas, or identified as pathogen vectors, have been investigated as targets for biological control using entomopathogenic microorganisms [7]. Others are also ideal models for genetic research due to features such as small genomes, short life cycles, and fewer ethical constraints compared to vertebrate systems. These characteristics support the investigation of microbial virulence mechanisms and the evaluation of novel antibiotic formulations.

The coevolution between insects and bacteria stems from long-term coexistence, immune plasticity, and strong environmental influences, forming a dynamic system that shapes insect physiology, adaptation, and ecological success [8,9,10]. Over hundreds of millions of years, insects and their bacterial partners have developed highly specialized relationships that influence feeding, development, immunity, and behavior. Many insects rely on obligate endosymbionts for essential nutrients, while others maintain diverse gut microbial communities that vary with diet, life stage, and habitat [11]. These associations are deeply rooted in evolutionary history and have contributed to the extraordinary ecological dominance of insects [12].

Although insects lack the antibody-based adaptive immunity of vertebrates, they mount rapid and efficient cellular and humoral immune responses. Infection outcomes in both insects and mammals often involve enzymatic cascades and conserved signal transduction pathways. These shared features are considered evolutionarily conserved defensive strategies rather than fully equivalent immune systems [13,14,15]. In particular, studies in *Drosophila melanogaster* have identified key signaling pathways underlying humoral and cellular defenses that share core elements with vertebrate innate immunity, providing a useful framework for understanding general principles of host–pathogen interactions [16,17]. In addition, insects such as *Galleria mellonella* and *Bombyx mori* are widely used as model organisms to study host–microbe dynamics, virulence, antimicrobial activity, and infection clearance [18,19]. Findings from these insect models can inform antibiotic efficacy studies and support translation into preclinical research using conventional animal models. A central feature of insect–microbe interactions is immune plasticity, meaning the ability of insects to finely regulate immune responses in order to tolerate beneficial microbes while defending against pathogens. The insect immune system actively shapes gut microbiota composition and stability through antimicrobial peptides (AMPs), reactive oxygen species (ROS), and immune signaling pathways, maintaining a balance between microbial symbionts and immune defense. At the same time, microbial variation can itself generate phenotypic plasticity in insects. Differences in gut microbiomes influence host physiology, behavior, and ecological interactions, showing that microbes actively contribute to insect traits rather than acting as passive passengers. These host–microbe relationships are strongly influenced by environmental factors such as diet, temperature, habitat, and chemical exposure. Environmental conditions determine which microbes insects acquire and maintain, while microbial communities in turn shape how insects respond to ecological pressures. Recent research highlights the complexity of the host–microbe–environment triad [20], demonstrating that microbial communities shift with environmental change and can mediate insect responses across trophic levels. Together, insect–bacteria coevolution, immune plasticity, conserved immune mechanisms, and environmental influences form an integrated framework for understanding how insects adapt, thrive, and interact within ecosystems.

## 2. Insect Immunity: A Brief Overview

### 2.1. Routes of Pathogen Entry and Immune Defenses in Insects

**Figure 1:**
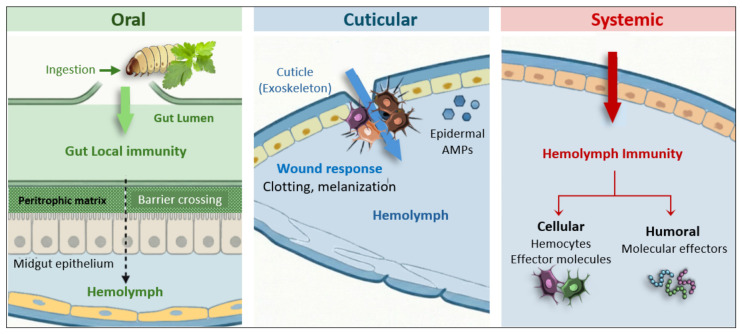
Infection routes and immune barriers. Oral: ingested pathogens face gut local immunity but can sometimes cross the peritrophic matrix and midgut epithelium, reaching the hemocoel cavity. Cuticular: breaching the exoskeleton triggers wound responses (clotting, melanization) and epidermal AMPs. Systemic: direct entry into the hemolymph activates cellular defenses and humoral effectors.

In insects, each potential route of infection can trigger distinct local or systemic immune responses (Figure 1). Oral infection occurs through ingestion, where pathogens encounter gut immunity in the lumen, including ROS and reactive nitrogen species (RNS), AMPs, and digestive enzymes. The peritrophic matrix and midgut epithelium act as main physical barriers, and only pathogens that successfully cross them reach the hemolymph. Cuticular infection results from breaches in the exoskeleton, triggering local wound responses such as clotting and melanization, along with the production of epidermal AMPs, before pathogens can access the hemolymph.

However, the immune response in fat body tissues and hemocytes can be triggered before pathogens enter the hemolymph, starting as early as their entry into the gut or adhesion to the cuticle. Systemic infection directly exposes the hemolymph to pathogens, activating both cellular and humoral immunity [21,22,23]. Although direct systemic exposure is uncommon in natural conditions, where penetration can occur through the body natural opening, it is a standard method in experimental assays, occurring after pathogens have been ingested or administered via microinjection into the hemocoel [21,24].

### 2.2. Key Signaling Pathways

Insect immunity is mediated exclusively by innate mechanisms that detect and respond to invading microorganisms through conserved molecular signals [25]. Central to this process is pathogen recognition via interactions between pathogen-associated molecular patterns (PAMPs) and host pattern recognition receptors (PRRs). PRRs include soluble hemolymph proteins and membrane-associated receptors such as peptidoglycan recognition proteins (PGRPs), lipopolysaccharide-binding proteins (LBPs), and other microorganism-recognition factors (Table 1) [26].

**Figure 2:**
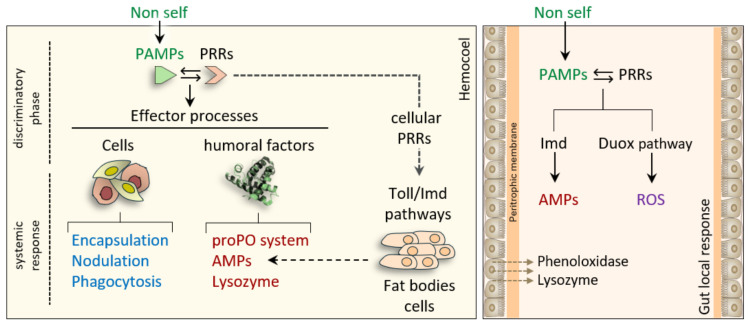
Systemic and local immune responses in insects. The interaction between PAMPs and PRRs triggers systemic immune responses in the hemocoel and local responses in the gut. Immune processes include cellular defenses, such as phagocytosis, encapsulation, and nodulation, and humoral ones, such as AMPs production, melanization by the prophenoloxidase–phenoloxidase (proPO) system, lysozyme activity, and activation of dual oxidase (Duox) pathway with ROS production.

While it is often presented as a highly specific detection system, the degree of immune specificity in insects is still debated, particularly with respect to how host receptors discriminate between pathogens versus triggering broadly reactive responses that prioritize tolerance and energetic efficiency over precision. Although immune pathways are well characterized (Figure 2), their relative importance, regulatory architecture, and activation thresholds vary across insect orders. For example, the number and diversity of PGRP genes, AMPs repertoires, and pathway cross-regulation differ markedly between Diptera, Lepidoptera, Coleoptera, and Hymenoptera.

**Figure 3:**
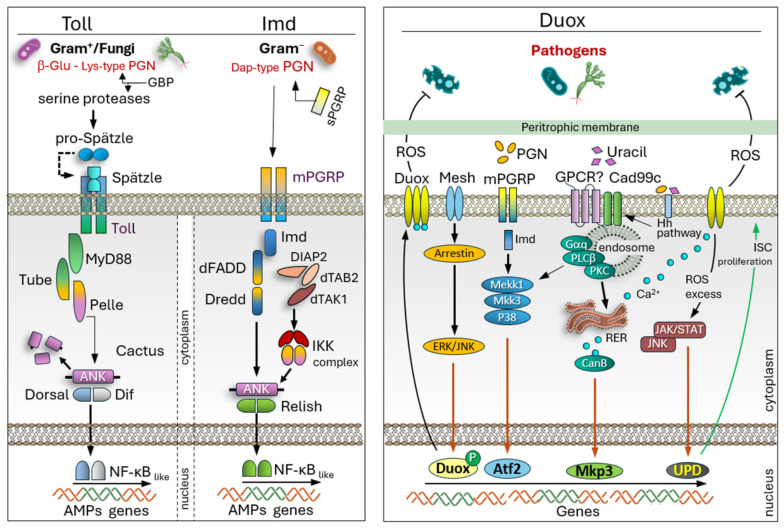
Schematic representation of the Toll, Imd and Duox signaling pathways in *D. melanogaster*. The pathways are activated by the presence of bacterial PAMPs. Intracellular signal transduction leads to the activation of transcription factors that enter the nucleus and turn on the genes encoding AMPs or ROS production.

These receptors trigger downstream signaling primarily through the Toll and Imd pathways, which regulate transcriptional immune responses via NF-κB-like transcription factors (Figure 3) [27]. The Toll pathway is activated by fungal β-glucans and Gram-positive bacteria through PRRs such as glucan binding proteins (GBPs) and PGRPs, leading to Toll receptor engagement at the plasma membrane. In the cell cytoplasm, the adaptor complex MyD88, Tube, and Pelle transduces the signal, resulting in phosphorylation dependent degradation of the IκB-like inhibitor Cactus. This releases the NF-κB-like transcription factors Dorsal and Dif, which translocate into the nucleus and induce transcription of AMPs genes, whose transcripts are key elements in bacterial clearance [28].

The Imd pathway is triggered primarily by Gram-negative bacterial peptidoglycan (PGN), recognized by membrane-bound PGRP-LC and cytosolic PGRP-LE. Signal transduction involves Imd, dFADD, and caspase Dredd, along with the TAK1, TAB2 complex and DIAP2, culminating in the activation of the IKK complex. Relish, a NF-κB-like factor, is phosphorylated and cleaved, allowing its N-terminal Rel domain to enter the nucleus and drive AMP genes expression [29].

The gut immune system functions as an integrated network in which multiple signaling pathways work together to maintain a balance between the effective elimination of pathogens and tolerance towards commensal microorganisms. The interaction between the Imd, ROS, Jak/Stat pathways and the prophenoloxidase–phenoloxidase (proPO) system ensures a rapid yet controlled response, preventing excessive immune activation that could injure host tissues. The gut microbiota itself plays a crucial role in modulating these immune responses, influencing signaling pathways and contributing to resistance to colonization by invading pathogens. A disruption of this delicate balance, whether due to environmental stress, pathogen invasion or dysbiosis, can lead to impaired immune function and increased susceptibility to infection. Consequently, understanding the coordination between immune signaling, epithelial renewal and microbial interactions in the insect gut provides valuable insights into host–microbe relationships. Insect tissues respond to bacterial invasion by triggering acute inflammatory reactions, highlighting the constant challenge faced by mucosal epithelia exposed to diverse microbial communities.

Recent studies across multiple animal models have demonstrated a dynamic, bidirectional interaction between the gut microbiota and the host immune system: the immune system shapes microbial composition, while resident microbes influence immune function and responsiveness [30]. In this context, the dual oxidase (Duox)–Mitogen-Activated Protein Kinase (MAPK)-Jak/Stat signaling axis (Figure 3) plays a central role in gut immunity, as it is activated by tissue damage and promotes epithelial regeneration during infection [31]. Microbiota-derived metabolites can influence host signaling pathways involved in epithelial renewal, stem cell proliferation, and immune tolerance, highlighting the intimate molecular dialog between symbiotic microbes and the insect immune system [32,33].

Genetic studies in *Drosophila*, along with analyses of gut-associated bacteria with varying capacities to stimulate the Duox pathway, have provided insight into mechanisms underlying homeostatic inflammation and chronic inflammatory conditions [34]. Furthermore, impairment of Duox function or its regulator Socs36E reduces infection tolerance, particularly in male dipterans, emphasizing the importance of ROS in host defense [35]. Figure 3 shows the Duox pathway activated in gut epithelial cells in the presence of pathogens [36]. When pathogens enter the gut environment, they release molecular signals like PGN and uracil. These molecules are detected at the cell surface by specialized receptors, including membrane-bound mPGRPs and possibly G protein-coupled receptors (GPCRs), while proteins such as Cad99c contribute to signal organization and internalization processes. After recognition, multiple intracellular signaling cascades are triggered. Detection of PGN activates the Imd pathway, which in turn initiates a kinase cascade involving Mekk1, Mkk3, and p38. This signaling influences transcription factors such as Atf2, promoting the expression of immune-related genes. In parallel, GPCR activation stimulates a Gαq-PLCβ-PKC pathway that increases intracellular calcium levels. The rise in Ca^2+^ is a key step, as it directly activates the Duox enzyme, enabling it to produce ROS. Additional regulatory layers refine this response. Arrestin-mediated processes modulate receptor activity and connect to ERK and JNK signaling pathways, ensuring proper signal strength and duration. At the same time, ROS generated by Duox serve not only as antimicrobial agents but also as signaling molecules. When ROS levels become high, they activate stress-responsive pathways such as JNK and Jak/Stat. These pathways coordinate protective responses and help maintain tissue integrity. At the transcriptional level, these combined signals converge to regulate genes including Duox itself, as well as Atf2, Mkp3, and UPD. The UPD cytokine activates Jak/Stat signaling, which promotes intestinal stem cell proliferation and supports tissue repair. Overall, the Duox pathway represents a tightly controlled defense mechanism in which pathogen detection leads to ROS production, immune gene activation, and regenerative signaling, ensuring both protection against infection and restoration of epithelial homeostasis [34]. The integration of studies on insect immunity have revealed that the innate response is regulated through interactions with metabolism, microbial symbiosis, epigenetic mechanisms, and systemic communication between organs. These studies suggest that immune activation is closely linked to metabolic reprogramming at the molecular level [37,38]. Conserved pathways such as Toll, Imd, and JNK not only regulate the expression of AMPs but also modulate lipid and carbohydrate metabolism to support the energy demands of immune responses [39,40]. During infection, metabolic intermediates and mitochondrial ROS also act as signaling molecules, influencing the transcription of immune genes and cellular stress responses [41]. Epigenetic regulation represents another important level of immune control. Histone acetylation and methylation, along with chromatin remodeling, regulate the accessibility of immune-related genes during exposure to pathogens. Recent works suggest that infection-induced metabolic changes may alter the activity of epigenetic enzymes, thereby linking cellular metabolism to the transcriptional regulation of immunity [42,43]. Furthermore, non-coding RNAs, particularly microRNAs (miRNAs) and long non-coding RNAs (lncRNAs), contribute to post-transcriptional regulation by targeting components of the Toll, Imd, and Jak/Stat pathways, precisely regulating AMP production, apoptosis, and antiviral responses [44,45].

Also, systemic immune regulation is based on inter-tissue signaling between gut, fat bodies, hemocytes, endocrine organs, and nervous system. Cytokine-like molecules, insulin-like peptides, and hormonal mediators coordinate immune activation with developmental and metabolic processes, ensuring body homeostasis during infection [46]. Taken together, these findings have expanded our understanding of innate immunity in insects, revealing a highly integrated network in which molecular immune signaling is closely linked to metabolism, microbiota composition, epigenetic plasticity, and the physiological regulation of the whole organism.

### 2.3. Molecular and Cellular Effectors

**Figure 4:**
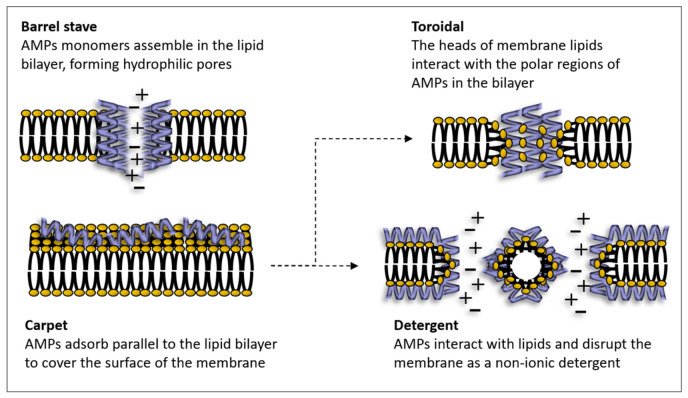
Models of the mechanisms of action of α-helical AMPs.

The main key components of bacterial clearance, the AMPs, include anionic and cationic molecules such as defensins, cecropins, drosomycin, drosocins, attacins, diptericins, melittin, etc., described in various insect species [47,48]. The short cationic AMPs exert an antimicrobial effect by destabilizing the microbial membrane. The positive charge, hydrophobic property, and secondary structure of peptides are essential for their antibacterial activity. The main mechanisms directed to the bacterial membrane are the Barrel stave, Toroidal and Carpet models. The last is an intermediate model that generates Toroidal pores or detergent action (Figure 4).

The formation of pores induces a drastic modification of the membrane permeability that progressively destabilizes the membrane structure. In the Barrel-stave model, AMPs insert into the membrane and form stable channels.

In the Toroidal pore model, both peptides and lipid groups form transient pores across the membrane. In the Carpet model, peptides accumulate on the membrane and compromise its integrity, leading to the formation of Toroidal pores or detergent-like membrane disintegration. AMPs damage the bacterial membrane causing a loss of membrane potential through disrupted ionic flux regulation [49].

An additional mechanism of action of AMPs involves their internalization into cells, where they act at multiple levels by disrupting membrane septum formation and inhibiting cell wall synthesis, nucleic acid synthesis, protein synthesis, and enzymatic activity. AMPs induction shows substantial interspecific variation in expression levels, inducibility, and spectrum of activity, suggesting that immune effectiveness is shaped not only by qualitative differences but also by dose and timing of AMPs formation [48,49].

Besides AMPs, humoral defenses include lysozymes and the proPO system [50,51]. This enzymatic system mediates melanization responses that contribute to pathogen immobilization and elimination, but it may also pose risks of self-damage due to cytotoxic intermediates [52]. This has fueled debate on how tightly melanization is regulated across taxa and ecological contexts, and whether its functional parallels with the vertebrate complement system reflect deep homology or convergent solutions to similar immune challenges [53]. Cellular immunity, mediated by hemocytes, includes phagocytosis, encapsulation, and nodulation [54,55,56]. The prevalence and efficiency of these responses vary with hemocyte abundance and composition, which differ widely between insect species and developmental stages. Such variability can limit the use of insect models as direct proxies for vertebrate immunity, particularly for studying immune memory, clonal expansion, or fine-scale pathogen discrimination [57]. At mucosal interfaces, such as in the intestine, immune responses must maintain a delicate balance between the effective elimination of pathogens and tolerance toward the commensal microbiota [58]. Local activation of the Imd pathway, together with the production of ROS mediated by Duox, modulates local responses [59]. However, the regulatory thresholds distinguishing immune activation from tolerance remain poorly characterized.

### 2.4. Immune Priming in Insects

An issue of interest, still under debate in insect immunology, is the potential presence of a form of immune memory. This aspect is relevant for understanding the interactions between insects and bacterial pathogens. In this context, several studies have described a phenomenon referred to as immune priming, whereby prior exposure to a sublethal dose of a pathogen enhances the host subsequent immune response. As a consequence, primed individuals often exhibit increased resistance to later infections with lethal doses of the same pathogen [60,61]. This process is often associated with expanded hemocyte populations and increased production of AMPs. Across studied systems, a common theme is that immune memory varies between insects but is consistently shaped by similar immune mechanisms. Priming does not rely on a single effector but rather results from a coordinated modulation of cellular and humoral processes and is adapted to the pathogen encountered. Unlike other insects, *Manduca sexta* does not possess a stable gut microbiome; however, it has been observed that protection against *Photorhabdus luminescens* is primarily mediated by PRRs, which trigger a broad humoral response [62]. The lack of a stable microbiome in some insects [63,64,65,66] could make them a useful experimental model for analyzing immune responses in the absence of potential interference from gut-resident bacteria. In contrast, *Tribolium castaneum* displays exceptional fine-scale discrimination, developing protection only against the specific *Bacillus thuringiensis* genotype previously encountered [67,68], while remaining unprotected against other bacterial strains. Social insects such as *Bombus terrestris* display an intermediate strategy, maintaining long-lasting, species-specific protection against *Paenibacillus* spp. and *Pseudomonas fluorescens* [61]. Transgenerational priming in *Tenebrio molitor* is conferred not through persistent infection or inherited immune transcripts, but via the targeted deposition of a restricted set of AMPs into the egg, enhancing resistance to *Staphylococcus aureus*, *B. thuringiensis*, and *Serratia entomophila* in offspring [69,70]. Insect priming immunity is thus a spectrum of memory-like states varying in specificity, duration, and cost, shaped by the pathogen type, prior exposures, and ecological context, rather than a simple on-off mechanism or vertebrate-like adaptive response.

## 3. Bacterial Strategies to Overcome the Host Immune Responses

### 3.1. Bacterial Evasion/Depression Strategies

**Figure 5:**
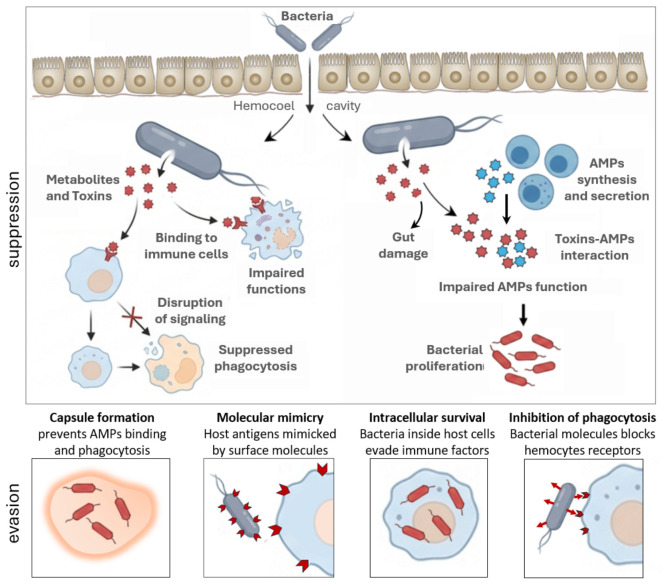
Bacterial immunosuppressive/evasive strategies. After penetration, bacteria can reach the insect hemocoel, where they encounter systemic immune defenses. Bacteria can act by releasing toxins, which neutralize the immune system. Toxins interact with immune cells, interfering with signaling pathways and impairing cellular functions. The action of AMPs can be impaired by toxins, resulting in bacterial proliferation. Toxins that damage the intestinal epithelium facilitate bacterial translocation into the hemocoel. Mechanisms implemented by bacteria to evade the insect immune response include strategies such as the formation of protective capsules (biofilms), penetration into host cells (intracellular bacteria), the action of enzymes that degrade humoral factors, or recognition evasion mechanisms.

Across insect–bacteria associations, a striking convergence emerges between entomopathogenic bacteria and free-living pathogens in the deployment of immunosuppressive strategies that target conserved components of insect innate immunity. Regardless of whether bacteria are delivered directly into the hemocoel by entomopathogenic nematodes or enter via oral ingestion, successful colonization depends on the ability to evade or suppress host immune recognition and modulate downstream effector responses (Table S1) [3,18,62,64,68,71,72,73,74,75,76,77,78,79,80,81,82,83,84,85,86,87,88,89,90,91,92,93,94,95,96,97,98,99,100,101,102,103,104,105,106,107,108,109,110,111,112,113,114,115,116,117,118,119,120,121]. The effectiveness of bacterial pathogenesis is often attributable to the action of bacterial metabolites and toxins (Figure 5 and Table 2). Although toxins are key elements in immune modulation, they are not exclusive. Other factors, such as metabolic competition, nutrient sequestration, growth rate, and spatial niche occupancy, can contribute significantly to pathogenesis [122,123].

Central to this process is the interference with PRR-mediated pathways, which represent major hubs of AMPs induction in insects. Pathogenic bacteria can enter the insect through injuries in the cuticle or through oral ingestion, releasing toxins that interfere with multiple components of the insect immune response. The toxins bind to immune cells, interfering with signaling pathways and compromising cellular functions such as phagocytosis. At the same time, AMPs activity [47] can be reduced by their interactions with toxins, resulting in decreased AMPs and bacterial proliferation [124,125]. The toxins also damage the intestinal epithelium, facilitating further translocation of bacteria into the hemocoel and compromising the immune function of the insect host.

Both obligate entomopathogens (e.g., *Photorhabdus* spp. and *Xenorhabdus* spp.) and free-living entomopathogens such as *Serratia* spp., *Pseudomonas* spp., and *B. thuringiensis* converge on similar outcomes: inhibition of NF-κB-like transcription factors, suppression of AMPs expression, impairment of hemocyte-mediated defenses, and disruption of epithelial barrier integrity (Figure 4 and Figure 5) [72,100,126,127,128]. These strategies add to the evasion/suppression mechanisms observed in different species, underscoring their evolutionary conservation. Moreover, the possible formation of biofilms, intracellular persistence, modification of surface antigens, and expression of virulence traits regulated by sensing threshold further increase resistance to immune clearance (Figure 5).

An additional important aspect of bacterial biology involves the mechanisms governing the transition from pathogenicity to mutualism. During the evolutionary shift from parasitism to mutualistic association, bacteria typically exhibit reduced virulence alongside increased host benefits, particularly when vertical transmission aligns bacterial fitness with that of the host [129]. This transition is frequently associated with genomic reduction, whereby symbionts lose genes required for autonomous survival while retaining functions essential for host support. Some examples of this process have been documented: in taxa such as *Sodalis* spp. and *Hamiltonella defensa*, an evolutionary transition from parasitic ancestors to endosymbiotic states has been proposed, indicating that host–microbe relationships exist along a continuum rather than as discrete categories of pathogen or symbiont [130,131]. The interaction between *Aphis fabae* and *H. defensa* illustrates this functional plasticity: the bacterium confers protection against parasitoid wasps (e.g., *Aphidius* spp.) yet imposes fitness costs in environments lacking such natural enemies [132]. Similarly, the system involving *Sodalis praecaptivus* and *Oryzaephilus surinamensis* demonstrates how novel symbionts can invade hosts, establish vertical transmission, and rapidly displace ancestral symbionts such as *Shikimatogenerans silvanidophilus* [133]. Together, these examples underscore the context dependency of microbial effects on host biology.

**Table 2:** Bacterial toxins and secondary metabolites.

Bacteria	Compounds	Tx, SM, AMP	Target Structure	Mechanism of Action	Insect Orders	Ref.
*Bacillus* *thuringiensis*	Cry toxins Cyt toxins Vip toxins Sip toxins Aerolysin β	Tx	Midgut epithelial receptors	Pores formation Pores formation Apoptosis Cytotoxic	Lepidoptera, Diptera, Coleoptera	[134,135]
*Lysinibacillus* *sphaericus*	Binary toxin (BinA/BinB)	Tx	Midgut epithelial receptors	Pores formation	Diptera	[136]
*Paenibacillus* *larvae*	Paenilamicin	SM	Midgut epithelia	Cytotoxic	Hymenoptera	[137]
*Photorhabdus* *luminescens*	Tc toxins Mcf toxin Pir toxins Pit toxins	Tx	Cytoskeleton Hemocytes Midgut cells Gut cells	Cytoskeleton damage Apoptosis Pore formation Cytotoxic	Lepidoptera, Diptera, Coleoptera	[138]
*Xenorhabdus* spp.	Xenocoumacin Xenematides Xenortides Benzalacetone Rhapidosin	SM	Immune cells Signaling	Antibiotic Immunosuppressive Immunosuppressive PLA2 inhibition Cytotoxic	Many orders	[139]
*Serratia* spp.	Sep complex ShlA hemolysin Serratamolide	Tx Tx SM	Midgut cells Cells membrane Cells membrane	Gut damage Membrane lysis Membrane surfactant	Coleoptera Many orders	[140,141]
*Pseudomonas* spp.	Monalysin FitD Fit-like	Tx	Gut epithelia Hemocytes Hemocytes	Pore formation Immune suppression Cytotoxicity	Diptera Lepidoptera	[142,143,144]
*Clostridium* *bifermentans*	Mosquito Tx	Tx	Midgut epithelia	Gut cell lysis	Diptera	[145]
*Yersinia* spp.	Yen Tx complex Tc-like Tx	Tx	Cytoskeleton Cytoskeleton	Cell disruption	Lepidoptera	[146,147]
*Chromobacterium* *subtsugae*	Violacein	SM	Cellular respiration	Oxidative stress	Lepidoptera, Diptera	[148]
*Burkholderia* spp.	Thailandamide Bongkrekic acid	SM	FA synthesis Mitochondria	Metabolic inhibition ADP/ATP transport	Many orders	[149,150]
*Streptomyces* spp.	Avermectins Spinosyns Milbemycins	SM	Nervous system	Cl^−^ channel activation Nervous stimulated Paralysis	Many orders	[151,152,153]
*Brevibacillus* *laterosporus*	BL Laterosporulin	Tx AMP	Midgut epithelia Cell membranes	Gut damage Membrane damage	Many orders	[154,155]

Tx: toxin, SM: secondary metabolites, AMP: antimicrobial peptide, FA: fatty acids.

### 3.2. Balancing Virulence and Symbiosis in Insect–Microbe Systems

**Figure 6:**
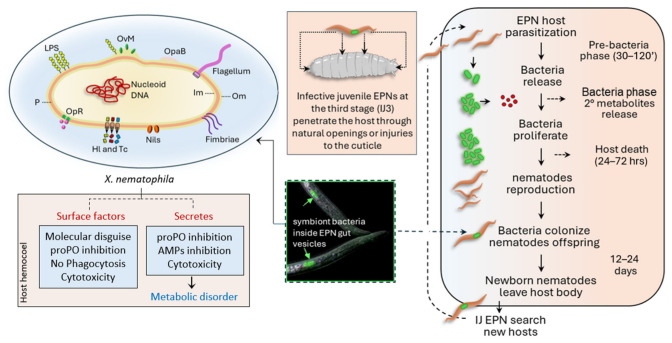
Infective entomopathogenic nematode (EPN) life cycle and its symbiont *Xenorhabdus nematophila.* The bottom panel illustrates the cellular structure of *X. nematophila*, highlighting key components involved in virulence, motility, host interaction, and survival, including lipopolysaccharides (LPS), outer membrane proteins (e.g., Omp/Opa), fimbriae, flagella, secretion systems, and other surface-associated factors. OvM: outer membrane vesicles; OpaB: cell surface adhesin; Om: outer membrane; Im: inner membrane; Nils: nematode colonization membrane proteins; Hl: hemolysin; Tc: toxin complex; OpR: oligopeptides receptors; P: periplasm.

The trade-off between virulence and symbiosis is evident in various bacteria, such as the genera *Photorhabdus* and *Xenorhabdus* [126,127], whose success depends on their ability to effectively kill the host insect while maintaining long-term compatibility with their nematode partners (Figure 6). Nematodes and their bacterial partners have a peculiar life cycle: the infective entomopathogenic nematodes (EPNs) at the third larval stage (IJ3) penetrate the insect host through natural openings or cuticular lesions and release their symbionts into the hemocoel from nematode intestine vesicles. The bacteria proliferate rapidly in the hemolymph, producing secondary metabolites responsible for evading/suppressing the host immunity, causing the death of the target insect within 24–72 h. Symbiotic bacteria also release antibiotic compounds to prevent colonization by other microorganisms [156,157]. Excessive virulence destabilizes the ecological niche required for nematode development and bacterial transmission, whereas insufficient immune suppression limits bacterial proliferation in the host hemocoel [158]. Bacterial transcription factors, such as Lrp, enable symbionts to modulate gene transcription in response to environmental signals, thereby finely regulating the shift between virulent behavior and cooperative interactions with the nematode [127]. In this context, immunosuppression is not purely destructive but rather modulatory, finely tuned to enable rapid exploitation of the host while preserving resources within the insect corpse.

**Figure 7:**
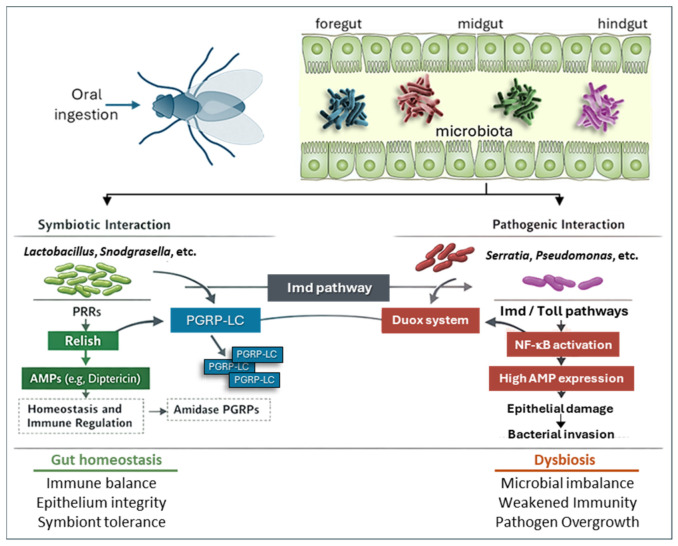
Gut immune responses regulate symbiosis and pathogenesis in insects. Bacteria entering the gut through oral ingestion encounter the resident microbiota. Symbiotic bacteria (e.g., *Lactobacillus* spp., *Snodgrassella* spp.) are recognized by PRRs, such as PGRP-LC, which triggers the Imd pathway, leading to the synthesis of AMPs, as well as the action of the PGRP amidase, which maintains immune tolerance and intestinal homeostasis. In contrast, pathogenic bacteria (e.g., *Serratia* spp., *Pseudomonas* spp.) trigger marked activation of the Imd and Toll pathways, Duox system, and the expression of AMP genes, causing epithelial damage and dysbiosis.

Similar interactions are also observed in gut-associated bacteria, where controlled activation of immune pathways by symbionts sustains tolerance and homeostasis (Figure 7), while dysregulation or loss of microbial diversity shifts interactions toward pathogenicity. Microorganisms enter the insect gut through oral ingestion and encounter resident microbiota along the intestinal epithelium. Symbiotic bacteria (e.g., *Lactobacillus* spp., *Snodgrassella* spp.) recognized by PRRs, including PGRP-LC, lead to controlled activation of the Imd pathway, Relish signaling, and basal AMPs production, as well as the action of amidase PGRPs to maintain immune tolerance and gut homeostasis. In contrast, pathogenic bacteria (e.g., *Pseudomonas* spp., *Serratia* spp.) trigger strong activation of the Imd and Toll pathways, Duox activation, and high AMPs expression, which can cause epithelial damage, bacterial invasion, and dysbiosis [72,128]. The balance between these responses is essential for maintaining intestinal integrity and immune homeostasis (Figure 7).

**Figure 8:**
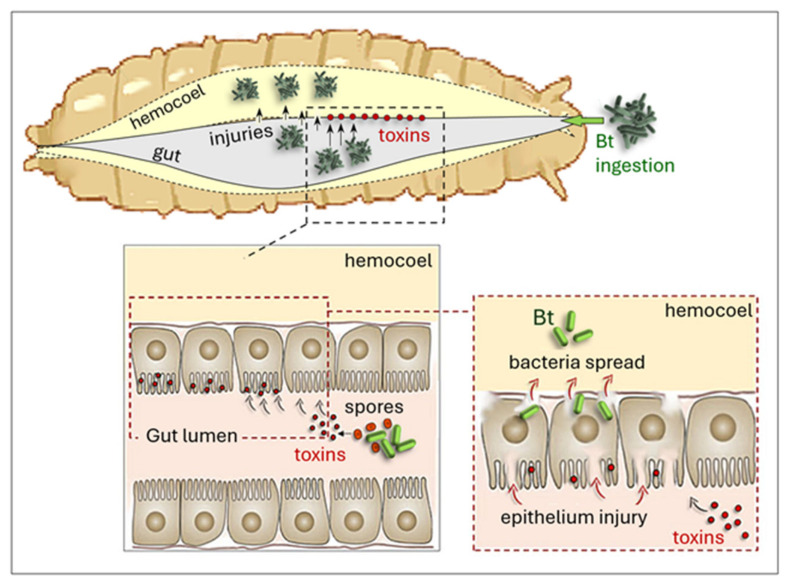
Schematic representation of *B. thuringiensis* (Bt) toxin activity in the host gut. After ingestion, Bt spores and toxins are released into the gut lumen, where the toxins damage the intestinal epithelium and compromise barrier integrity. This disruption allows bacteria to cross into the hemocoel, leading to systemic infection and extensive tissue damage.

Thus, immune modulation emerges as a continuum rather than a binary state, with bacterial fitness determined by the ability to optimize host survival (i.e., to be long enough to ensure transmission). Bacterial toxins emerge as a unifying mechanism driving immune evasion across diverse insect–bacteria interactions. Although toxins differ in structure, targets, and delivery routes, their functional convergence is evident in their capacity to simultaneously damage tissues and suppress immune defenses (Figure 5). Pore-forming toxins (e.g., Cry, Tc, Pir), apoptosis-inducing factors (e.g., Mcf), and enzymatic toxins targeting cytoskeletal or signaling components collectively dismantle both cellular and humoral immunity by inducing hemocyte apoptosis, blocking phagocytosis, inhibiting melanization, and compromising AMPs activity (Table 2) [124]. For instance, following ingestion of *B. thuringiensis*, bacterial spores and toxins are released into the gut lumen, where toxins disrupt the intestinal epithelium, causing cellular damage and barrier dysfunction. Gut injuries facilitate the translocation of bacteria and toxins across the epithelium into the hemocoel, leading to systemic infection and widespread tissue damage (Figure 8) [45].

Lethal infection in a variety of lepidopterans often requires a tripartite interaction in which Cry toxins perforate the midgut, allowing resident gut commensals to spread and cause mortality [125,159]. Notably, toxin-mediated immune evasion is not restricted to highly specialized symbionts but is equally prominent in free-living entomopathogens such as *B. thuringiensis* and *Lysinibacillus* spp., indicating strong selective pressure that favors this strategy [100,101]. Even in gut-associated infections, where immune activation is robust, toxins can overwhelm local defenses by disrupting epithelial integrity and enabling bacterial translocation into the hemocoel. This convergence highlights toxins as multifunctional effectors that integrate virulence with immune modulation rather than acting solely as cytolytic agents [100].

Taken together, insect–bacteria interactions reveal a conserved toolkit of immunosuppressive strategies shaped by evolutionary trade-offs between exploitation and coexistence. Toxin-mediated immune evasion processes, the presence of common mechanisms that modulate changes in virulence, and the finely tuned regulation within the gut highlight how insect immunity has played a powerful selective role [24,160]. Viewing entomopathogenic and symbiotic bacteria through this comparative lens not only clarifies common principles of host–microbe interactions but also promotes insects as valuable models for understanding the evolution of immune evasion strategies across biological systems.

## 4. Use of Insect–Bacteria Models to Deepen Their Interactions

In addition to offering insights into host–pathogen interactions during natural infections, insects serve as valuable alternative in vivo models for investigating experimental infections. Direct systemic infection is widely employed in laboratory settings and often yields significant data, even if this method may not faithfully replicate the outcomes observed in natural infections. These trials represent a valuable approach for analyzing pathogen–host interactions or testing new antimicrobial compounds [161,162]. Among the insect models commonly used in these laboratory tests are *D. melanogaster* [163], *B. mori* [164], and *G. mellonella* [6,102], which are genetically versatile and easily manipulable systems, as well as *Apis mellifera*, which is of particular importance in studies of social immunity and transmission of inherited microbiomes [164,165,166].

Integrated multi-omic approaches have improved our understanding of insect–microbe interactions and immune regulation at the molecular level. Previous research, based on single-omic datasets, often failed to capture the complexity of interactions between insect hosts, microbial symbionts, pathogens, and environmental stressors. The integration of transcriptomics, metagenomics, metabolomics, proteomics, epigenomics, and single-cell technologies now enables analysis at the level of immune signaling, microbial homeostasis, and host physiological adaptation [167,168].

A number of insect models have been extensively investigated using integrated omics approaches to elucidate the molecular basis of host–microbe interactions and immune regulation. In *D. melanogaster*, commensal bacteria such as *Lactobacillus plantarum* and *Acetobacter pomorum* regulate intestinal stem cell proliferation, ROS homeostasis, and epithelial renewal through Imd and JNK signaling pathways [169]. Similarly, in *Aedes aegypti* and *Anopheles gambiae*, microbiota members as *Wolbachia pipientis*, *Asaia bogorensis*, and *Enterobacter* spp. strongly modulate antiviral and antiparasitic immunity. In particular, *Wolbachia* limits Dengue, Zika virus, and *Plasmodium* replication through ROS-mediated immune priming and competition for host lipids required for pathogen replication [170,171]. In *B. mori*, infection with *B. thuringiensis* and *Serratia* spp. triggers broad transcriptional activation of melanization cascades, stress response genes, and AMPs production [170,171]. The honeybee *A. mellifera* represents another well-characterized model, hosting a relatively stable gut microbiota dominated by *Gilliamella apicola*, *Snodgrassella alvi*, *Bifidobacterium asteroides*, and *Lactobacillus* spp., which contribute to nutrient metabolism, pathogen resistance, and immune homeostasis [164,172].

Metagenomic and metatranscriptomic analyses have further expanded the understanding of insect-associated microbial functional potential. Shotgun sequencing has revealed microbial gene repertoires involved in vitamin biosynthesis, amino acid metabolism, quorum sensing, biofilm formation, and detoxification processes [173,174]. In termites such as *Macrotermes natalensis* and *Reticulitermes flavipes*, gut symbionts including *Treponema*, *Fibrobacter*, and methanogenic archaea encode cellulases, hemicellulases, lignin-degrading enzymes, and nitrogen-fixation pathways that enable lignocellulose digestion and nutrient acquisition. These consortia also produce acetate, the primary energetic substrate for the host [175,176]. Overall, integrated omics approaches consistently highlight microbial contributions to host metabolism, immune modulation, and pathogen susceptibility. In mosquitoes, as mentioned before, *Wolbachia* and *Asaia* alter lipid metabolism and immune gene expression, thereby influencing vector competence, while bacteria such as *Serratia odorifera* can increase susceptibility to arboviral infection by affecting intestinal barrier integrity and immune suppression [177,178]. Multi-omics integration further supports bidirectional host–microbe communication mediated by coordinated regulation of bacterial virulence factors and insect immune pathways [179].

Metabolomics has become essential for understanding the biochemistry of these interactions. Immune-related metabolites, including short-chain fatty acids, sphingolipids, polyamines, eicosanoids, and indole derivatives, have been shown to regulate oxidative stress responses and antimicrobial defenses [180,181]. In honeybees, microbial metabolites produced by *Gilliamella* and *Lactobacillus* participate in carbohydrate fermentation, amino acid biosynthesis, and detoxification of plant-derived phytochemicals [182]. In arbovirus-infected mosquitoes, metabolic remodeling of phosphatidylcholine, triglycerides, and cholesterol pathways is tightly associated with viral replication efficiency and vector competence [183].

Beyond metabolic shifts, the molecular landscape of these interactions is further fine-tuned at the protein level. Post-translational modifications (PTMs) are essential for regulating the strength and duration of innate immune responses by modulating the activity and stability of signaling components. Phosphorylation and dephosphorylation serve as primary mechanisms for activating immune signaling pathways, while ubiquitination controls the turnover of key regulators to prevent uncontrolled inflammation. Additionally, modifications such as glycosylation play a vital role in the maturation and intracellular trafficking of pattern recognition receptors (PRRs), which is necessary for the effective detection of invading pathogens [184]. Moreover, significant variations in phenoloxidase activity, lysozyme-like enzyme levels, and circulating hemocyte counts are consistently observed, with the timing and magnitude of these responses depending on the specific immune challenge [185]. Secretome analyses have identified extracellular vesicles (EVs) and exosome-like particles as ubiquitous mediators of communication. These vesicles serve as carriers for proteins, lipids, and regulatory RNAs involved in infection, defense, and intercellular immune signaling [186].

Epigenomic regulation adds a layer of complexity to host–microbe interactions. DNA methylation, histone modifications, and chromatin remodeling, collectively shape the transcriptional landscape of immune-related genes and microbial tolerance mechanisms [187,188]. In eusocial insects such as honeybees, epigenetic regulation, including DNA methylation and histone modifications, is closely linked to caste differentiation, behavioral plasticity, and environmental adaptation. These mechanisms respond to external cues, such as social context or seasonality, to establish diverse phenotypes and longevity without altering the underlying genotype. Comparative studies of chromatin accessibility and transcriptome landscapes, particularly in the brain, have identified tissue-specific gene expression patterns that underpin social communication and task specialization [189,190]. Moreover, microbial metabolites can directly influence histone acetylation and methylation states, thereby connecting microbial metabolism to host epigenetic regulation.

Single-cell omics technologies have provided unprecedented resolution in dissecting immune cell heterogeneity. Single-cell RNA sequencing (scRNA-seq) in *D. melanogaster* and *A. aegypti* has identified distinct hemocyte subpopulations specialized for phagocytosis, melanization, clotting, and antiviral defense [191,192]. Canonical markers such as hemolectin, nimrod, lozenge, and prophenoloxidase are widely used to define differentiation states within the insect immune system [191,193]. The integration of scRNA-seq with spatial transcriptomics and single-cell ATAC-seq enables a comprehensive analysis of molecular dynamics, linking chromatin accessibility to gene expression and revealing tissue organization at subcellular resolution. These multi-omic approaches are transformative for mapping immune responses and understanding the mechanistic basis of host–microbe interactions [192,194], revealing strong spatial coupling between microbiota distribution, epithelial renewal, and barrier immunity.

Improvements in computational biology and artificial intelligence have significantly accelerated the integration of heterogeneous omics datasets. Graph neural networks, systems biology models, and machine learning approaches are increasingly being used to reconstruct host–microbe regulatory networks and identify molecular nodal points associated with immune resilience or susceptibility to pathogens [195]. These integrated analyses highlight central signaling axes, including Relish, FOXO, JNK, and TOR, which coordinate immunity, metabolism, and stress responses across multiple levels of biological organization [196].

Integrated multi-omics approaches are revolutionizing the molecular understanding of insect–microbe interactions by linking microbial community with host immune signaling, metabolism, and epigenetic regulation. The combination of transcriptomic, metabolomic, proteomic, and single-cell technologies now allows investigation of biological systems across multiple levels of organization, from molecular pathways to ecological interactions [197]. Future developments in spatial multi-omics, long-read sequencing, synthetic microbiome engineering, and causal network modeling are expected to further elucidate mechanisms underlying symbiosis, pathogen resistance, and immune adaptation in insects [198]. These advances may support the development of microbiome-based pest management strategies, improved vector control programs, and more sustainable agricultural practices [199].

### 4.1. Insect Infection with Gram-Negative Bacteria

Gram-negative bacteria represent a large and evolutionary diverse group of pathogens. As infection models, they provide a versatile platform for studying a variety of processes, from preliminary assessments of antibiotic efficacy to the investigation of virulence determinants and of the complex mechanisms underlying the transmission dynamics of enteric pathogens. A major strength of insect systems lies in their ability to reveal how infection route, host physiology, and ecological context influence disease outcomes [22].

Among these, larval hosts such as *G. mellonella* are widely used to identify virulence determinants, assess antimicrobial efficacy, and evaluate therapeutic strategies in vivo. Several studies on *Acinetobacter baumannii* indicate that this lepidopteran model can be used to assess the efficacy of antibiotics such as meropenem, cefotaxime, gentamicin, and tetracycline, which either target the cell wall or inhibit protein synthesis. Meropenem and gentamicin were shown to significantly increase the survival of *G. mellonella*, whereas cefotaxime and tetracycline, against which the bacterium is resistant, did not affect larval survival [74,75]. This insect also provides a valid and effective model for studying *Klebsiella pneumoniae* infections. Insua et al. [76,77] demonstrated that the larva can be used to study bacterial pathogenesis, as it is capable of distinguishing between pathogenic and non-pathogenic strains and of reproducing key features of infection, including cell death associated with bacterial replication, evasion of phagocytosis, and suppression of host immune responses. Mutants lacking the capsular polysaccharide or exhibiting alterations in lipid A or in the outer membrane proteins OmpA and OmpK36 displayed attenuated virulence, still eliciting host immune responses. Furthermore, the gene expression has been investigated revealing an upregulation of genes involved in capsule biosynthesis and Lipid A remodeling, under the control of the PhoPQ and PmrAB regulatory systems. In addition to these results, other studies have shown that *G. mellonella* infected with *K. pneumoniae* exhibits virulence traits comparable to those observed in mammalian models, supporting its use as a model for evaluating antimicrobial strategies [76,77].

Studies on motile pseudomonads in *D. melanogaster* reveal that systemic injection causes acute virulence by interfering with chemosensory signaling of the Pil/Chp system, whereas oral exposure triggers a melanization response that traps *Pseudomonas aeruginosa* in a persistent, antibiotic-tolerant state [78,79]. *D. melanogaster* can also be used to monitor intestinal colonization during polymicrobial infections caused by *Aeromonas* spp. [81]; the authors designed an infection method using multiple *Aeromonas* species to distinguish the effects of polymicrobial mixtures. Individual strains killed more than 80% of the flies in 20 h and the course of infection was altered with microbial mix.

The versatility of these insect models also extends to studying complex intracellular pathogenesis and high-impact diseases, as demonstrated by the effectiveness of wax moth larvae as a new model for *Helicobacter pylori* infection [82]. *G. mellonella* larvae were inoculated with different wild-type *H. pylori* strains or mutants lacking virulence determinants, such as the VacA toxin, and the associated factors CagA and CagE, the entire cag pathogenicity island (PAI), urease, and gamma-glutamyl transpeptidase (GGT). The authors found that both wild-type and mutant *H. pylori* strains were able to survive and replicate in *G. mellonella* larvae. Mutant strains were less virulent than their respective wild type. In addition, larval death was consistently associated with the induction of apoptosis in hemocytes. This lepidopteran also exhibits similar hallmarks of infection as observed in mammals, such as the intracellular replication of *Brucella* spp. and *Legionella pneumophila* [83,200]. *Brucella* caused long-lasting infections when the lipopolysaccharide (LPS) and envelope lipids were modified, reducing PAMP recognition and thereby hindering the activation of innate immunity. Compared to *K. pneumoniae*, the tested *Brucella* spp. induced a delayed and less severe mortality profile, consistent with evasion of detection by immunity. Strains of *L. pneumophila,* isolated from various environments, which exhibit variability in virulence-related genes, display different levels of pathogenicity in *G. mellonella* [83,200]. *M. sexta* larvae are used as a model organism to investigate the pathogenesis and virulence of *Pseudomonas aeruginosa*, an opportunistic pathogen in both humans and insects. *M. sexta* is especially useful for studying highly virulent bacteria and analyzing microbial virulence factors rigorously: strains of *P. aeruginosa* injected directly into the hemocoel resist phagocytic encapsulation and proliferate extensively in the hemolymph [80].

Furthermore, non-conventional models such as the German cockroach (*Blattella germanica*) highlight how host behaviors, including coprophagy, and gut microbiota composition, influence the horizontal transmission of enteric pathogens and the hosts immune resistance to infections like *Salmonella enterica* [84,85,86]. In this species, the gut microbiota exerts a protective role against *S. enterica*: rather than competing with bacterial growth in feces, commensal bacteria promote the expression of AMPs that suppress infection. Finally, neither the total abundance of the microbiota nor its overall diversity appears to correlate with susceptibility to infection.

### 4.2. Insect Infection with Gram-Positive Bacteria

Parallel to Gram-negative systems, insect-based models have become increasingly important for elucidating the molecular mechanisms underlying Gram-positive bacterial pathogenicity. Gram-positive bacteria such as *Enterococcus* spp., *Staphylococcus* spp., *Bacillus* spp., *Listeria monocytogenes*, *Micrococcus luteus*, and *Streptococcus* spp. have been extensively investigated using invertebrate hosts, enabling detailed characterization of virulence determinants and host immune responses.

To investigate the contribution of specific virulence determinants, including the *fsr* quorum-sensing system and gelatinase activity, *Enterococcus* spp. have been evaluated using *G. mellonella* as an in vivo infection model [103]. *Enterococcus faecalis* strains, including those of dairy origin, exhibit strong larvicidal activity comparable to the reference strain OG1RF, whereas *E. durans* and *E. faecium* display attenuated or avirulent phenotypes. Targeted deletion of *fsrB* and *gelE* genes results in gelatinase-negative phenotype, confirming disruption of enzyme production. Functional analyses demonstrate that both Δ*fsrB* and Δ*gelE* mutants exhibit reduced virulence in *G. mellonella*, with a more pronounced attenuation observed in Δ*fsrB* strains, indicating that *fsrB* regulates additional virulence-associated pathways beyond gelatinase production.

With regard to *S*. *aureus*, insect models have elucidated immune evasion mechanisms at the molecular level. D-alanylation of wall teichoic acids (TA) reduces activation of the Toll-mediated humoral response in *D. melanogaster* by impairing PGN recognition by the host receptor PGRP-SA, thereby promoting bacterial persistence [104]. Complementary studies in *T*. *molitor* using cell-free systems demonstrate that purified D-alanylated WTA loses its inhibitory effect when dissociated from PGN, indicating that immune evasion depends on the covalent linkage between WTA and PGN rather than on higher-order cell wall structure [105]. The increasing number of microorganisms that are resistant to antibiotics is prompting the development of new antimicrobial compounds and strategies to fight bacterial infections. The use of insects to screen and test new drugs is increasingly considered a promising tool to accelerate the discovery phase and limit the use of mammalians. The silkworm *B*. *mori* has been used as an in vivo system to test glycopeptide antibiotics against *Staphylococcus epidermidis*, where treatments with vancomycin, teicoplanin, and dalbavancin significantly improve larval survival, reduce mortality, and limit excessive immune activation [106].

*D. melanogaster* and *Culex* spp. have been employed to investigate the activity of anthrax toxins produced by *Bacillus anthracis*. In these systems, the protective antigen PA_83_ is cleaved into PA_20_ and PA_63_. Notably, PA_20_ enhances host resistance to infection independently of bacterial growth inhibition by interacting with PGRP-SA and activating the Toll/NF-κB signaling pathway. This suggests a role for host immunity in modulating anthrax pathogenesis [107].

Further insights into Gram-positive intracellular and systemic infections have been obtained using *L*. *monocytogenes*. *G. mellonella* enables comparative virulence analyses across multiple *Listeria* species, including non-pathogenic (*L. innocua*, *L. seeligeri*, *L. welshimeri*) and pathogenic (*L. ivanovii*) strains, revealing dose- and strain-dependent virulence differences without significant variation between serotypes [108]. Moreover, studies in *M. sexta* with *Streptococcus pneumoniae* demonstrate that unencapsulated strains such as R6 retain virulence and can induce mortality, suggesting that capsule-independent mechanisms contribute to pathogenicity in insect hosts [109]. Infections with *M. luteus* in *G. mellonella* have established a standardized low-virulence model, with defined growth dynamics and an LD_50_ of about 10^4^ CFU per larva, providing a reproducible framework for assessing host immune efficiency and enabling comparative analyses of infection dynamics [110].

Overall, insect models offer a unified and complementary platform for studying both Gram-negative and Gram-positive bacteria, bridging molecular mechanisms of virulence with host immune responses and supporting the development of innovative antimicrobial strategies.

## 5. Conclusions

Insect–bacteria interactions are dynamic evolutionary systems shaped by continuous interplay between host immunity, microbial adaptation, and ecological factors. Studies in insect models have revealed conserved innate immune pathways, mechanisms of immune modulation, and the importance of microbiota in maintaining host homeostasis. These interactions show that bacterial virulence depends not only on toxins, but also on metabolic flexibility, immune evasion, and niche adaptation, while insect defenses rely on coordinated cellular, humoral, and microbiota-mediated responses. Despite major advances, important questions remain regarding immune specificity across insect taxa, the regulation of immune tolerance, and the ecological consequences of microbiota disruption. Current knowledge is still focused on *D. melanogaster* and *G. mellonella*, highlighting the need for broader comparative studies across insect lineages.

Recent developments in multi-omic approaches, single-cell transcriptomics, spatial imaging, and microbiome analyses are providing increasingly detailed insights into host–microbe interactions. Together with standardized polymicrobial infection models and experimental evolution studies, these approaches will improve understanding of how immunity, metabolism, and microbial communities interact under natural conditions.

Beyond their ecological relevance, insects are valuable experimental systems for studying bacterial pathogenicity, antimicrobial resistance, host tolerance, and novel antimicrobial compounds, while also supporting the development of sustainable biocontrol strategies. Overall, future progress will depend on integrating ecology, immunology, microbiology, and systems biology to better understand the complexity and broader significance of insect–bacteria interactions.

## Figures and Tables

**Table 1:** Main bacterial PAMPs and insect PRRs.

Bacterial PAMPs	Description	Insect PRRs	Immune Pathway
Peptidoglycan (Lys-type)	Wall component mainly Gram-positive	PGRP-SA, PGRP-SD	Toll pathway
Peptidoglycan (DAP-type)	Wall component Gram-negative, some Gram-positive	PGRP-LC, PGRP-LE	Imd pathway
Lipopolysaccharide (LPS)	Outer membrane component Gram-negative	PGRP-LC GNBP3 proteins	Indirect recognition via PG fragments
Lipoteichoic acid (LTA)	Gram-positive wall	GNBP1 with PGRP-SA	Toll pathway
Flagellin	Structural protein of flagella	Some lectins and unidentified PRRs	Not well characterized
Bacterial DNA (CpG motifs)	Unmethylated CpG-rich DNA	Intracellular sensors (not well defined)	Not well characterized
β-1,3-glucan (fungi, bacteria)	Cell wall polysaccharide	βGRPs proteins	Toll pathways

## Data Availability

No new data were created or analyzed in this study. Data sharing is not applicable to this article.

## Supplementary Materials

The following supporting information can be downloaded at: https://www.mdpi.com/article/10.3390/insects17050515/s1, Table S1: Bacteria and Insect host: infection routes and effects.

## Abbreviations

The following abbreviations are used in this manuscript:

PAMPs	Pathogen-associated molecular patterns
PRRs	Pattern recognition receptors
PGRPs	Peptidoglycan recognition proteins
PGN	Peptidoglycan
GNBPs	Glucan-binding proteins
LBPs	Lipopolysaccharide-binding proteins
SBPs	Sugar-binding proteins
NF-κB	Nuclear factor kappa-light-chain-enhancer of activated B cells
AMPs	Antimicrobial peptides
proPO	Prophenoloxidase–Phenoloxidase
ROS	Reactive oxygen species
RNS	Reactive nitrogen species
EPNs	Entomopathogenic nematodes
TA	Teichoic Acid
CFU	Colony-Forming Unit
